# Protection of early phase hepatic ischemia-reperfusion injury by cholinergic agonists

**DOI:** 10.1186/1472-6890-6-3

**Published:** 2006-02-15

**Authors:** Elahé T Crockett, James J Galligan, Bruce D Uhal, Jack Harkema, Robert Roth, Kinnari Pandya

**Affiliations:** 1 Departments of Physiology & Division of Human Pathology, College of Human Medicine, Michigan State University, East Lansing, USA; 2 Department of Pharmacology, College of Human Medicine, Michigan State University, East Lansing, USA; 3 Department of Pathology, College of Veterinary Medicine, Michigan State University, East Lansing, USA

## Abstract

**Background:**

Cytokine production is critical in ischemia/reperfusion (IR) injury. Acetylcholine binds to macrophages and inhibits cytokine synthesis, through the cholinergic anti-inflammatory pathway. This study examined the role of the cholinergic pathway in cytokine production and hepatic IR- injury.

**Methods:**

Adult male mice underwent 90-min of partial liver ischemia followed by reperfusion. The AChR agonists (1,1-dimethyl-4-phenyl-L-pioperazinium-iodide [DMPP], and nicotine) or saline-vehicle were administered *i.p. *before ischemia. Plasma cytokine tumor necrosis factor (TNF)-α, macrophage inflammatory protein-2, and Interleukin-6 were measured. Liver injury was assessed by plasma alanine transaminase (ALT) and liver histopathology.

**Results:**

A reperfusion time-dependent hepatocellular injury occurred as was indicated by increased plasma-ALT and histopathology. The injury was associated with marked elevation of plasma cytokines/chemokines. Pre-ischemic treatment of mice with DMPP or nicotine significantly decreased plasma-ALT and cytokines after 3 h of reperfusion. After 6 h of reperfusion, the protective effect of DMPP decreased and reached a negligible level by 24 h of reperfusion, despite significantly low levels of plasma cytokines. Histopathology showed markedly diminished hepatocellular injury in DMPP- and nicotine-pretreated mice during the early-phase of hepatic-IR, which reached a level comparable to saline-treated mice at late-phase of IR.

**Conclusion:**

Pharmacological modulation of the cholinergic pathway provides a means to modulate cytokine production and to delay IR-induced heaptocellular injury.

## Background

The central nervous system (CNS) and the immune system are involved in functionally relevant cross-talk, which functions to maintain homeostasis [1]. Recent studies have indicated that the CNS has an anti-inflammatory role in preventing the development of lethal systemic inflammation [2-4]. For example, pro-inflammatory mediators (e.g., TNF, IL-1) activate afferent pathways in the vagus nerve, which stimulates the release of the pituitary adrenocortical hormone; the resultant increase in plasma corticosteroids suppresses cytokine release to prevent excessive inflammation [3,4]. In addition to this sensory function of the vagus nerve in systemic inflammation, there exists an efferent or motor vagus neural mechanism by which acetylcholine (ACh) inhibits cytokine release from resident tissue macrophages [5]. Studies have shown that direct electrical stimulation of the efferent vagus nerve resulted in significant inhibition of the development of endotoxin-induced shock and aortic clamping-induced inflammatory response during IR [5,6]. The inhibitory effect was associated with decreased levels of the pro-inflammatory cytokine, TNF [5,7]. It appears that CNS-derived motor output through the cholinergic anti-inflammatory pathway modulates the development of systemic inflammation, which is referred to as the " inflammatory reflex" [2].

Hepatic IR is a feature of many clinically important scenarios, including hepatic surgery, transplantation, trauma, and hemorrhagic shock [8]. This injury occurs in a biphasic pattern: The acute injury phase (early phase), which is characterized by liver injury occurring within 1–6 h after reperfusion, is associated with Kupffer cell activation, release of the pro-inflammatory cytokines, and generation of reactive oxygen species (ROS) [8-10]. This phase is followed by the subsequent subacute-phase response (late phase), which is characterized by a massive neutrophil infiltration and further production of the inflammatory mediators. It has been shown that inhibition of Kupffer cell function by gadolinium chloride results in diminished cytokine production and hepatic IR injury [11]. Additionally, cytokine neutralization by antibodies significantly attenuated liver injury induced by hepatic IR [12,13], suggesting that control of cytokine regulation can serve as an important target to limit inflammatory IR injury and death.

The aim of the current study was to examine the role of the cholinergic anti-inflammatory pathway in cytokine production and liver injury following hepatic IR. It was of particular interest to determine whether pharmacological stimulation of this pathway through nicotinic receptors would protect against liver IR injury in mice. Acetylcholine, the principal neurotransmitter of the vagus nerve, binds to nicotinic and muscarinic receptors on macrophages. As such, two acetylcholine receptor (AChR) agonists were applied in a model of partial liver IR in mice. The inflammatory response and tissue injury were evaluated by plasma cytokines and ALT levels, and histopathology. The data demonstrated that pretreatment of mice with the nicotinic AChR agonists significantly inhibited cytokine production during early and late phases of IR, while the protective effect on hepatocellular injury was significant primarily during the early phase of liver IR.

## Methods

All chemicals were purchased from the Sigma Chemical Co. (St. Louis, MO), unless otherwise noted.

### Experimental animal model of hepatic ischemia and reperfusion

All animals received humane care in compliance with the Guide for the Care and Use of Laboratory Animals (National Institutes of Health Publication No. 85–23, revised 1985). Experimental protocols were reviewed and approved by the Michigan State University Animal Use and Care Committee. Adult (8–10 wk) male C57BL/6 mice (Charles River Laboratories, Portage, MI) weighing between 24–29 g were fed a standard diet and acclimated in the animal housing area for 1 week before experimentation. The mice were randomly divided into two groups. The test group underwent IR, while the sham group underwent the same surgical protocol but without vascular occlusion, to induce IR. Each group was further subdivided into three groups, receiving either DMPP, nicotine or vehicle (i.e., saline). Following treatment, a partial hepatic IR model was applied. This model induces severe ischemic insult to the liver without inducing hypertension and subsequent bacterial translocation into the portal venous blood [14]. The mice were lightly anesthetized with inhaled methoxyflurane (Baxter Caribe, Inc., Guayama, PR), followed by an *i.p. *injection of 35 mg/kg sodium pentobarbital (Abbott Laboratories, North Chicago, IL). A midline laparotomy was performed, the portal circulation to the median and left lateral lobes of the liver was carefully dissected, and an atraumatic vascular clip (Accurate Surgical and Scientific Instruments Corp. Westbury, NY) was placed on the vessels, interrupting the portal venous and hepatic arterial blood supply to these lobes. Five drops of sterile saline were applied over the abdominal viscera to keep the organs moist and compensate for the loss of fluid. The abdomen was temporarily closed with sterile staple sutures to prevent dehydration and possible contamination. The animal was kept in the recovery room under close supervision. After 90 min of partial hepatic ischemia, the clamp was removed and reperfusion was resumed. The abdomen was closed in a double layer using 5–O nylon, and 0.8 ml sterile lactated Ringer's Solution (Abbott Laboratories, North Chicago, IL) was administered subcutaneously to compensate for operative fluid loss. During the reperfusion, the mice were kept in clean cages with no further administration of anesthesia or analgesics. After reperfusion, mice were euthanized, and blood and tissue samples were collected, as described below.

All the surgical procedures were performed under aseptic conditions. In this model, the caudate and right lateral lobes, as well as the papillary and quadrate processes, retained an intact portal and arterial inflow and venous outflow to prevent intestinal venous congestion. This resulted in the induction of ischemia to approximately 70% of the liver. There was no to minimal bleeding during the surgical operation (bleeding <1+ of scale of 0–5+ considered minimal). If a small (2+) to moderate (3+) amount of bleeding occurred due to blood vessel puncture, the animal was euthanized and no further experimentation was carried out. The bleeding scale (0 – 5+) was arbitrarily based on saturation of each cotton swab that held about 150–200 μl of blood. This was considered as 1+ bleeding. The liver IR model with 90 min of ischemia induces a severe, isolated, and reproducible liver injury with very minimal animal mortality within one week (i.e. 0–5%).

### Drug administration

All animals received an *i.p. *injection of the nonselective nicotinic AChR agonist, 1,1-dimethyl-4-phenyl L-pioperazinium iodide (DMPPI) (1 mg DMPPI/kg B.Wt.), or nicotine ([-]-1-methyl-2- [3-pyridyl]pyrrolidine), 15 min prior to the onset of ischemia, and repeated at 3 and 6 h of reperfusion. Saline-treated mice received an equal volume of vehicle (saline).

### Peripheral blood and tissue procurement

Blood samples were obtained from the right ventricle via a left anterior thoracotomy at the time of sacrifice. The blood was collected in a sterile syringe containing 50 μl of heparin (100 USP Units/ml), and centrifuged to separate the plasma. The plasma samples were stored at -70°C until use for cytokine and ALT assays. A portion of the ischemic and non-ischemic liver lobes were fixed in buffered 10% formalin, embedded in paraffin, and used for hematoxylin and eosin (H&E) staining. Another portion of ischemic and non-ischemic liver lobes were snap frozen in liquid nitrogen and stored at -70°C for Reverse- Transcription Polymerase Chain Reaction (RT-PCR). Further, an additional section of liver was frozen in optimal cutting temperature compound (OTC media) and stored at -70°C until use for cryosectioning and immunohistochemistry staining.

### Measurement of plasma alanine aminotransferase levels

The plasma ALT levels were determined spectraphotometrically, as previously described [14,15]. The ALT values are expressed in international units per liter (IU/L).

### Histopathology

H&E staining was performed on tissue sections prepared at 5-μm intervals from paraffin-embedded liver tissue. A pathologist (J.H.), blinded to the experimental conditions, examined the liver tissue sections.

### Plasma cytokine concentrations

Plasma TNF-α, IL-6 and MIP-2 were determined in a 96-well Nunc-Immuno microplate (VWR Scientific, Chicago, IL), using a sandwich enzyme-linked immunosorbent assay (ELISA) technique, as previously described [11]. The capture antibody was a polyclonal anti-mouse TNF-α, IL-6 or MIP-2 specific goat IgG (R&D Systems, Minneapolis, MN) and the detection antibody was a biotinylated polyclonal anti-mouse TNF-α, IL-6 or MIP-2 specific goat IgG, (R&D Systems). All plasma samples were tested in duplicate. The minimal detectable protein concentration was 20 pg/ml.

### Analysis of mRNA by RT-PCR

Total RNA from ischemic and non-ischemic liver tissue was extracted by utilizing the Ultraspec™-II RNA isolation system (Biotecx, Houston, TX). An aliquot of 2000 ng of total liver RNA was reverse transcribed to complementary DNA (cDNA) using the Genamp RNA PCR protocol (Perkin-Elmer, Norwalk, CT) with random hexamers. Using the primers for MIP-2, IL-6, and TNF, cDNA products were amplified by PCR. PCR was initiated by heating the reaction at 95°C for 15 min to activate the Hotstart DNA polyemerase. The mixture was amplified for a total of 40 cycles using a 3-step cycle process that began with melting at 95°C for 20 sec, annealing at 55°C for 75 sec, followed by extension at 72°C for 60 seconds. The final cycle was followed by 10 min of incubation at 72°C. Twelve microliters of each RT-PCR reaction were electrophoresed in a 2% agarose (FMC Bioproducts, Rockland, ME) gel and stained with ethidium bromide. Reverse transcription and PCR amplification of Glyceraldehyde 3-phosphate dehydrogenase (GAPDH), a housekeeping gene, was performed to verify equal loading of RNA and cDNA in the RT and PCR reactions, respectively. Products of RT-PCR reactions were photographed using Kodak gel imager. PCR primers used are as follows: MIP-2 sense, (5') – AGT TTG CCT TGA CCC TGA AGC – (3'), and MIP-2 antisense, (5') – TGA ACT CTC AGA CAG CGA GGC ACA – (3') to give a 196 base pair product ; IL-6 primer mix (Designed by Superarray Bioscience Corporation, Frederick, MD) to give a 178 base pair product ; TNF-alpha primer mix (Designed by Superarray Bioscience Corporation, Frederick, MD) to give a 205 base pair product ; GAPDH sense (5') -ACA GCC GCA TCT TCT TGT – (3') and GAPDH antisense (5') – CGT GAG TGG AGT CAT ACT GG- (3') to give a 200 base pair product (Macromolecular Structure Facility Michigan State University, East Lansing, MI).

### Analysis of mRNA by Real-Time PCR

Total RNA was isolated from the livers using Ultraspec™-II RNA isolation system (Biotecx, Houston, TX). Reverse Transcription was performed as described above. Real-Time PCR, using SYBR Green reagent (Applied Biosystems) was performed using Stratagne Mx3000P. All reactions were done in a 25 μl reaction in triplicate following the manufacturer's protocol. PCR parameters consisted of 40 cycles using a 3-step cycle process that began with melting at 95°C for 20 sec, annealing at 55°C for 75 sec, followed by extension at 72°C for 60 sec. The final cycle was followed by 10 min of incubation at 72°C. Real-Time PCR was performed using sham, saline, DMPP and Nicotine treated samples. Each of the liver samples were tested for TNF-α, IL-6 and MIP2 expression levels. A Housekeeping gene, GAPDH, was used to verify equal loading of RNA and cDNA in the RT and PCR reactions. The data is presented as threshold cycle (Ct), the cycle at which fluorescence was determined to be statistically significant above background signal contributed by the labeled oligonucleotides within the PCR reaction. The Ct is inversely proportional to the log of the initial copy number.

### Determination of hepatic neutrophil accumulation by immunohistochemistry

To confirm the presence of neutrophils in the liver tissue, immunohistochemical staining was performed using 5-μm thick liver tissue section. The primary antibody (clone 7/4, IgG2a) specific to mouse neutrophils (Cedarlane, Westbury, NY), the biotin-conjugated secondary antibody (PharMingen, San Diego, CA), and Vectastain avidin-biotin complex reagent and 3,3'-diaminobenzidine chromogen kits (Vector Laboratories, Inc., Burlingame, CA) were used as previously described in detail [11]. A matched antibody isotype immunoglobulin (IgG) was applied as a negative control antibody to monitor the anti-neutrophil antibody specificity. Tissue sections were counter stained with hematoxylin (Gill's formula, Vector Laboratories) and mounted with DAKO Mounting Media (DAKO Corp, Carpinteria, CA). The samples were examined using a Nikon light microscope interfaced with a spot 24-Bit Digital Color Camera.

### Statistical analysis

The data is expressed as means ± standard error of the mean. Comparisons between two groups were performed using an unpaired *t*-test. Comparisons between multiple groups and various time points were analyzed using ANOVA followed by a Fisher's PLSD post-hoc test. *P *≤ 0.05 was considered statistically significant. All data was analyzed using the *StatView version 5.0.1 software^© ^*for Windows.

## Results

### Effects of nicotinic AChR stimulation on hepatocellular injury

Our previous published work has shown that 90 min of hepatic ischemia followed by reperfusion caused hepatocellular injury in a time-dependent fashion, as demonstrated by plasma ALT level [11,14]. After 90 min of ischemia followed by 3 h of reperfusion, there was moderate to severe liver injury histologically that increased by 6 h of reperfusion, and this injury was associated with significant plasma ALT elevation. Therefore, in the current study the effect of DMPP on liver injury was evaluated after 3, 6, and 24 h of reperfusion.

**Figure 1:**
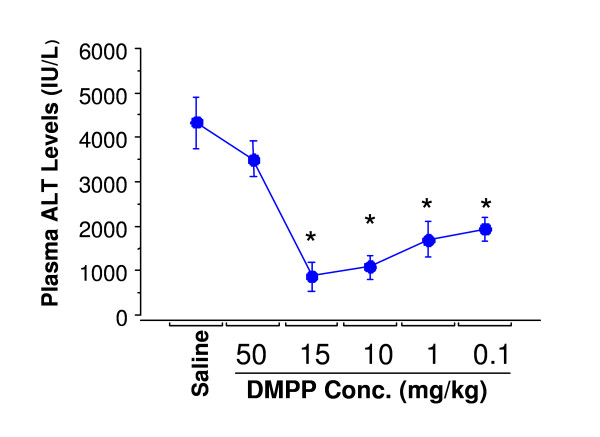
**Dose-response curve of AChR agonist inhibited hepatic IR-induced changes in plasma ALT levels**. Mice were pretreated with vehicle (i.e. normal saline), or DMPP 15 min prior to the onset of ischemia (90 min), followed by 3 h of reperfusion. Values are expressed as means ± SEM of *n *= 4 mice each group. *P < 0.05, Saline-treated group vs. DMPP-treated group.

**Figure 2:**
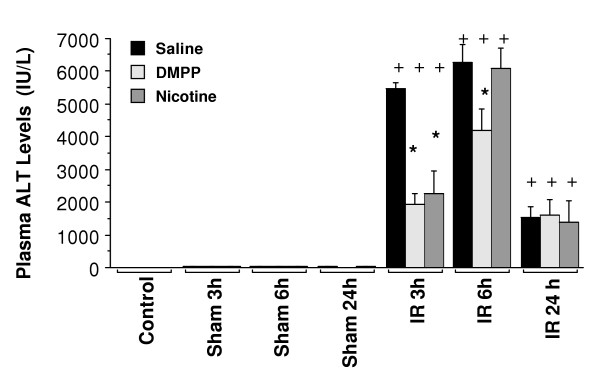
**AChR agonists inhibited hepatic IR-induced changes in plasma ALT levels**. Mice were pretreated with vehicle (i.e. normal saline), DMPP (1 mg/kg), or nicotine (1 mg/kg), prior to the onset of ischemia (90 min), followed by 3, 6, and 24 h of reperfusion (IR). Nicotine or DMPP treatment was administered 15 min prior to the onset of ischemia and repeated after 3 and 6 h of reperfusion. Sham animals received the same pretreatment as the IR group, followed by sham operation (n = 3 mice each group). "Control" group represents mice that received no drug treatment, and no surgical operation of IR/sham protocol was performed (n = 3 mice). Values are expressed as means ± SEM of *n *= 8 to 13 mice per saline or DMPP group, and n = 4–8 per nicotine group.**+***P *< 0.05, IR group vs. sham-operated group. *P < 0.05, Saline-treated IR group vs. DMPP-or nicotine-treated IR group.

In preliminary experiments the optimal dose of DMPP that produced no clinical signs (i.e., altered breathing, heart rate) and toxicity was determined. As Figure 1 depicts, plasma ALT levels in vehicle-treated mice significantly increased after 90 min of ischemia followed by 3 h of reperfusion. Pre-ischemic treatment of mice with DMPP significantly reduced ALT levels caused by 90 min of ischemia followed by 3 h of reperfusion. DMPP at 50 mg/kg body weight was toxic and caused immediate death of 60% of the animals due to respiratory paralysis and cardiac arrest (Figure 1). At 15 mg/kg, DMPP showed maximum protection from liver damage caused by IR with minor toxic effects. A dose of 1 mg/kg was determined to be optimal, and this concentration was applied in all the experiments presented in this study. This optimal dose was in the range that has been applied in previously published reports by other investigators [5,18]. There was no plasma ALT elevation in mice subjected to the sham operation indicating that neither DMPP nor the surgical procedures caused hepatic injury (Figure 2). A similar dose was found to be effective for nicotine (data not shown).

Subsequently, the effect of DMPP and nicotine on liver IR injury following various reperfusion times was examined. As Figure 2 shows, plasma ALT in mice treated with saline significantly increased after 3, 6, and 24 h of reperfusion as compared to the respective sham group. Preischemic treatment of mice with DMPP or nicotine significantly reduced ALT levels after 3 h of reperfusion compared to saline-treated mice (i.e., 65% inhibition for DMPP, and 69% for nicotine) (Figure 2). However, after 6 h of reperfusion, the protective effect of the agonists was significantly diminished. DMPP treatment resulted in a 34% decrease in plasma ALT levels, while nicotine had no effect (Figure 2). After 24 h of reperfusion, no significant inhibition of liver injury was observed upon either DMPP or nicotine treatment (Figure 2).

**Figure 3:**
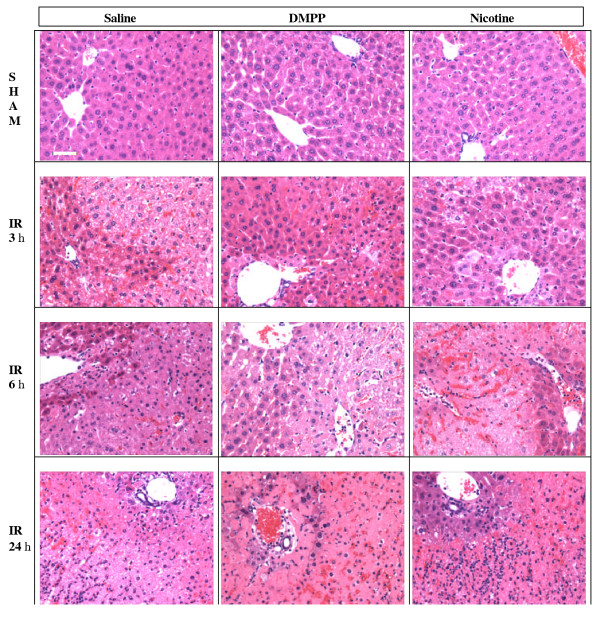
**Hepatic histopathology following IR**. Mice were pretreated with vehicle (i.e. saline), DMPP (1 mg/kg), or nicotine (1 mg/kg), prior to the onset of ischemia (90 min), followed by various reperfusion times (IR). Sham animals received the same pretreatment as the IR group, followed by sham operation. The ischemic liver sections were prepared and stained with H&E. The top row images represent the sham mice, which show normal hepatic histology. The following rows represent mice that were subjected to I/R. A pattern of reperfusion damage is evident by necrosis of hepatocytes in the pericentral and midzonal regions, with relative sparing of the periportal areas. Note the presence of neutrophils in the midzonal region around the central vein. Images are representative of n = 13 mice per saline or DMPP, and n = 8 per nicotine group.

Liver histopathology was evaluated based on sinusoidal congestion, cytoplasmic vacuolization, hepatocellular necrosis, and neutrophil infiltration. Administration of DMPP or nicotine did not cause any hepatocellular injury in sham-operated mice (Figure 3, top row), a result which correlates with negligible plasma ALT levels (Figure 2). Reperfusion of the ischemic liver caused hepatocellular necrosis and sinusoidal congestion by 3 h of reperfusion in saline-treated mice (Figure 3, IR 3 h). There was mostly sparing of the periportal areas with progressive injury in the midzonal and pericentral areas. In contrast, in DMPP- and nicotine-pretreated mice there were only minor patchy spots of mild necrosis in various areas of the liver tissue at 3 h. Most areas of the liver tissue from DMPP- and nicotine- treated mice exhibited normal structure similar to those of the sham group.

After 6 h of reperfusion of ischemic liver, there was extensive hepatocellular necrosis, sinusoidal congestion, and neutrophil infiltration in saline-treated mice (Figure 3, IR 6 h). Similarly, extensive hepatocellular injury was observed in the ischemic livers of DMPP- or nicotine-treated mice. Although not quantified, the heaptocellular damage and hepatic necrosis in DMPP-treated mice appeared less severe than those of the nicotine treated mice. After 24 h of reperfusion, liver histopathology exhibited similar pathology in all animals treated with saline, DMPP, or nicotine. Increased neutrophil infiltration was evident in the parenchyma of the liver for all three treatments. Because the inhibitory effects of DMPP and nicotine on liver injury significantly declined after 6 and 24 h of reperfusion, it was thought that perhaps these agonists were cleared from the body. Therefore, three additional doses of DMPP or nicotine were administered: one at the start of reperfusion, then repeated at 3 and 6 h of reperfusion. There were no significant differences compared to those mice which received only one dose of DMPP or nicotine (data not shown).

### Hepatic neutrophil accumulation

**Figure 4:**
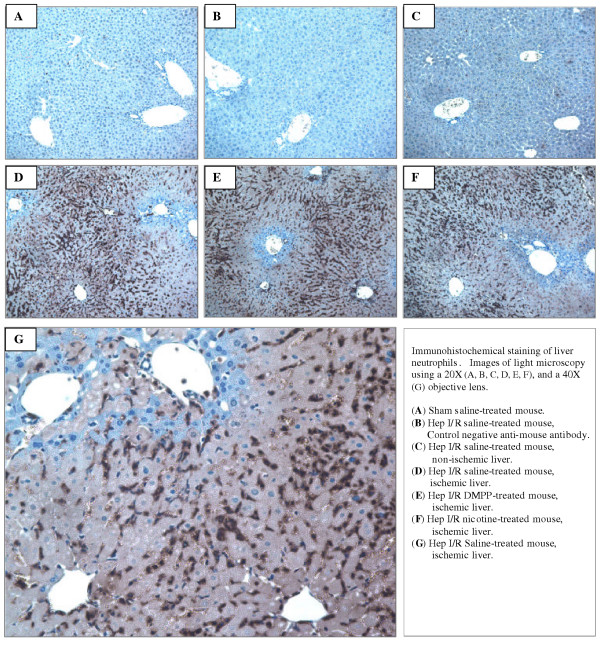
**Immunohistochemical staining of liver neutrophils**. Mice were pretreated with vehicle (i.e. normal saline), DMPP (1 mg/kg), or nicotine (1 mg/kg) prior to the onset of ischemia (90 min), followed by 24 h of reperfusion. Immunostaining of the liver sections was performed using a specific anti-neutrophil antibody. A matched antibody isotype IgG was used as a negative control antibody. Neutrophils are presented by a dark brown stain. Images are representative of three separate experiments with similar results.

H&E stained liver sections presented a marked neutrophilic infiltration into the liver after 6 and 24 h of reperfusion. Immunohistochemical staining was used to confirm the presence of neutrophils in the liver parenchyma. There was no appreciable neutrophil infiltration into the liver tissue of sham-operated mice (Figure 4.A). After 3 h of reperfusion, the number of neutrophils present in the liver remained insignificant. Similarly, DMPP- or nicotine-treated mice exhibited minimal neutrophil infiltration at 3 h of reperfusion (data not shown). However, after 6 h of reperfusion, a marked increase of neutrophil infiltration into the liver parenchyma was observed, with maximal infiltration occurring by 24 h of reperfusion (Figure 4.D, 4.E, 4.F). Neutrophil infiltration appeared to be similar in all three groups (i.e., saline-, DMPP-, and nicotine-treated mice) as shown in Figures 4.D, 4.E, and 4.F. There was minimal neutrophil infiltration into the non-ischemic lobes of the mice subjected to hepatic IR, which was similar to those of the sham-operated mice (Figure 4.C), indicating that ischemia was a cause of neutrophil infiltration. Figure 4.B shows a liver section from a saline-treated mouse subjected to IR stained with the control anti-mouse antibody indicating the specificity of the anti-mouse neutrophil antibody.

### Effects of nicotinic AChR stimulation on plasma TNF-α, IL-6 and MIP-2 levels

**Figure 5:**
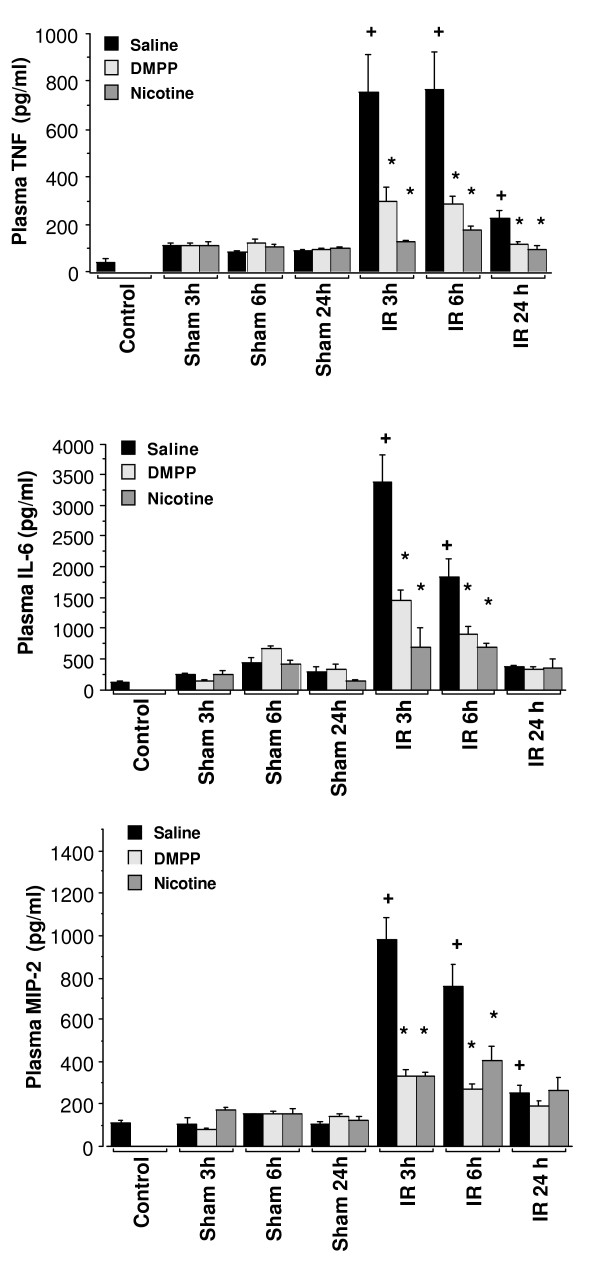
**AChR agonists effect on plasma cytokine induced by hepatic IR in mice**. Mice were pretreated with vehicle (i.e. normal saline), DMPP (1 mg/kg), or nicotine (1 mg/kg), prior to the onset of ischemia (90 min), followed by 3, 6, and 24 h of reperfusion (IR). Nicotine or DMPP treatment was initiated 15 min prior to the onset of ischemia (90 min) and repeated at 3 and 6 h of reperfusion. "Sham" animals received the same pretreatment as the "IR" group, followed by sham operation (n = 3 mice per group). "Control" group represents mice that received no drug treatment, and no surgical operation of IR/sham protocol was performed (n = 3 mice). Peripheral blood was collected, and plasma cytokines were measured using an ELISA. Values are expressed as means ± SEM (pg/ml) of *n *= 5 to 8 mice per group. **+***P *< 0.05, IR group vs. sham group. *P < 0.05, saline-treated group vs. DMPP-or nicotine treated group.

Inflammatory cytokines such as TNF-α and IL-6 have shown to play key roles in pathophysiology of hepatic IR injury [[12,13], and [16]]. In saline-treated mice, plasma concentrations of TNF-α and IL-6 were significantly increased after 90 min of ischemia followed by 3 and 6 h of reperfusion. This effect diminished by 24 h of reperfusion (Figure 5). Although the sham operation induced a slight cytokine response, plasma TNF-α and IL-6 in sham-operated animals remained low compared to the corresponding IR mice. Treatment of mice with DMPP or nicotine significantly reduced plasma levels of TNF-α at 3, 6, and 24 h of reperfusion (Figure 5). The treatment also significantly reduced plasma IL-6 after 3 and 6 h of reperfusion. This inhibitory effect on IL-6 was not significant after 24 h of reperfusion as the plasma level of this cytokine had already reached its baseline level (Figure 5). MIP-2, a CXC Chemokine, is a potent neutrophil chemoattractant, important in hepatic IR injury [12,13]. Similar to the other cytokines noted above, plasma chemokine MIP-2 level significantly increased in vehicle-treated mice after 3 and 6 h of reperfusion, then decreased by 24 h (Figure 5). Pre-ischemic treatment of mice with DMPP or nicotine significantly reduced the elevation in plasma MIP-2 caused by liver IR.

### Effects of hepatic ischemia-reperfusion on hepatic TNF-α, MIP-2 and IL-6 mRNA expression

**Figure 6:**
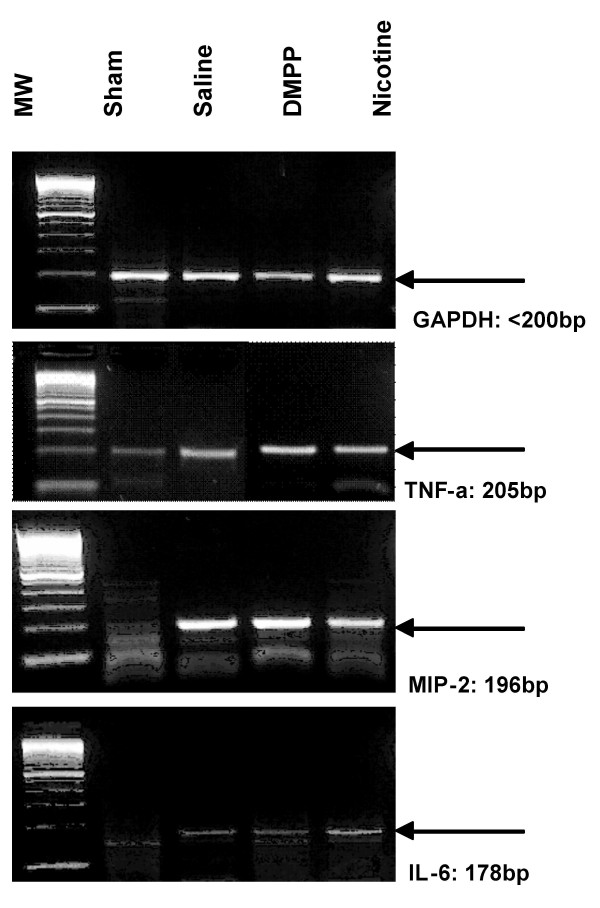
**Effect of Cholinergic agonists on hepatic expression of cytokine mRNA following IR**. RT-PCR analysis of TNF-**α**, MIP-2, and IL-6 mRNA liver from mice that were pretreated with vehicle (i.e. normal saline), DMPP (1 mg/kg), or nicotine (1 mg/kg), prior to the onset of ischemia (90 min), followed by 3 h of reperfusion (IR). The PCR products were run on 2.5% agarose gel, stained with ethidium bromide, and then visualized by ultraviolet illumination. GAPDH was used as a housekeeping gene. Blots shown are representative of four separate experiments with similar results.

**Figure 7:**
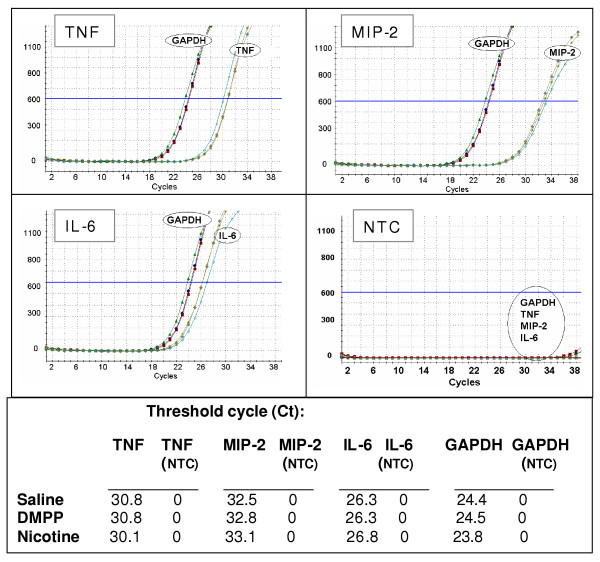
**Real-time RT-PCR**. Amplification of TNF-**α**, MIP-2, IL-6 and GAPDH cDNA in livers from mice that were pretreated with vehicle (i.e. normal saline), DMPP (1 mg/kg), or nicotine (1 mg/kg) prior to the onset of ischemia (90 min), followed by 3 h of reperfusion (IR). Results are expressed as fluorescence intensity of SYBR Green bound to DNA (Y-axis). The X-axis represents the number of cycles (i.e. 40 cycles). GAPDH was used as a housekeeping gene. NTC represents PCR products amplified in the absence of cDNA template. Graphs shown are representative of three separate experiments with similar results.

To investigate the mechanism of DMPP or nicotine inhibition on cytokine production, mRNA expression of the cytokines in ischemic liver tissue (3 h of reperfusion) was measured by RT-PCR and real-time PCR techniques. No appreciable levels of cytokine mRNA expression were observed in liver tissues of the matching sham-operated mice. However, reperfusion of the ischemic liver for 3 h resulted in an increase in mRNA expression of TNF-α, MIP-2 and IL-6, as shown in Figure 6. Treatment of mice with DMPP or nicotine did not significantly alter the mRNA expression, suggesting that the inhibitory effect was through a posttranscriptional mechanism (Figure 6). Further, RT real-time PCR analysis confirmed increased levels of TNF-α, MIP-2 and IL-6 mRNA, which were not significantly altered by DMPP or nicotine treatment. As shown in Figure 7, the threshold cycle (Ct) calculated at specific fluorescent intensity (i.e., relative DNA content) for TNF-α, MIP-2 and IL-6 remained unchanged in saline-, DMPP- or nicotine-treated liver samples. To ensure the specificity of the amplified DNA for TNFα, MIP-2, IL-6 and GAPDH, a no-template-control (Primer only, "NTC") was included for these primer sets. There was no amplification of PCR products, suggesting that the amplified cDNA for TNFα, MIP-2 and IL-6 were specific and not due to DNA contamination or rare non-specific secondary background (Figure 7, NTC).

## Discussion

The current study is the first to demonstrate the role of cholinergic anti-inflammatory pathway in hepatic IR injury. The findings showed that administration of nicotinic ACh agonists, DMPP and nicotine, significantly attenuated the release of cytokines/chemokine, and diminished the early phase of hepatic injury caused by liver IR in mice. In contrast, the late phase of liver injury was not protected, although cytokine/chemokine production was significantly decreased. These results highlight the differential mechanisms of reperfusion injury during early and late phases of IR.

The present data indicates an association between decreased cytokine production and hepatocellular injury during cholinergic agonist treatment. However, this association appears only during the early phase of liver IR. This finding supports previously published studies [5,7]. In an *in vitro *study, ACh significantly attenuated the release of pro-inflammatory cytokines (i.e., IL-1β, IL-6, and TNF) in LPS-stimulated human macrophage cultures [5]. In addition, direct electrical stimulation of the peripheral vagus nerve *in vivo *during lethal endotoxemia in rats inhibited TNF synthesis in liver, decreased plasma TNF levels, and prevented the development of shock [5]. TNF is the proximal cytokine generated during an inflammatory response that stimulates various cells including Kupffer cells and hepatocytes to produce various CXC chemokines such as MIP-2 and KC [16]. TNF has also been implicated in the production of other inflammatory mediators such as eicosanoids, nitric oxide and ROS, which can further exacerbates inflammation and tissue injury [17]. In hepatic IR, TNF is involved in the production of chemokines [12,16]. Previous studies by Colletti *et al. *[12,16] and Lentsch *et al. *[13] have shown that neutralization of TNF-α or MIP-2 by administration of antibodies significantly attenuated hepatocellular injury induced by hepatic IR. Further, our laboratory has previously shown that Kupffer cells were the major source of the cytokines and chemokines in liver IR, and inhibition of Kupffer cell activity significantly attenuated the liver IR injury [11]. Acetylcholine, the principal neurotransmitter of the vagus nerve, binds to nicotinic and muscarinic receptors on macrophages, and the receptor-ligand interaction inhibits the synthesis of TNF at a post-transcriptional level [5]. The nicotinic AChR α-7 subunit is essential for the inhibition of cytokine synthesis by the cholinergic anti-inflammatory pathway [18]. Therefore, the published literature and our data suggest that DMPP and nicotine interaction with AChR on Kupffer cells may possibly attenuate the synthesis of the pro-inflammatory cytokines as well as the development of liver injury during the early phase of liver IR in mice.

In contrast, there was no protective effect in the late phase of IR injury during cholinergic agonist treatment, as seen in the early IR injury phase. However, cytokine production was significantly attenuated in both early and late phases of IR injury. This finding does not support the findings reported by Wang *et al.*, who demonstrated that treatment of mice with nicotine provided lasting protection and not merely delayed the onset of death using a sepsis model [19]. It is interesting to note that in their study nicotine treatment did not have a significant effect on IL-6, which appears to be a marker in the prognosis in sepsis in patients and animals [20-22]. In addition, it was shown that protection against sepsis was attributed to the high mobility group box-1 (HMGB-1) protein, which was significantly diminished upon nicotine treatment. HMGB-1 is a late mediator of lethal systemic inflammation in sepsis [23], however, a recent study has suggested HMGB-1 as an early mediator of inflammation and organ damage in hepatic IR injury [24]. *In vitro*, necrotic cells as well as purified HMGB-1 trigger the production of pro-inflammatory cytokine TNF-α [25]. HMGB-1 is a monocyte-derived protein. It is also present in the nucleus of hepatocytes in the normal liver, which is significantly up-regulated after IR [24]. Whether the cholinergic agonists had any effect on HMGB-1 production in the study presented here remains unclear. The discrepancy between the effect of nicotine in providing long term protection against sepsis and not against liver IR injury may be attributed to the differences in the underlying pathophysiologic mechanisms of the two disease conditions.

Previous studies have shown a direct association between CXC chemokines, neutrophil recruitment and liver injury. Specifically, it has been shown that neutralization of CXC chemokines significantly attenuated neutrophil infiltration and liver injury in rat and mouse models of warm hepatic IR [12,13]. In support of these findings, the current study showed that inhibition of cytokine/chemokine production correlated with liver injury (plasma ALT level) during the early phase of liver IR injury. However, our study indicated that during the late phase of hepatic IR, cholinergic agonists significantly inhibited CXC chemokine production (i.e., MIP-2), which did not correlate with neutrophil infiltration and the liver IR injury. One explanation may be that the cholinergic agonist treatment did not sufficiently abolish CXC chemokine production and that the generated MIP-2 was able to trigger neutrophil infiltration and liver injury. Another explanation is that during the late phase of liver IR injury, cytokine/chemokine role may be of limited relevance for neutrophil infiltration and liver injury, suggesting participation of other more potent inflammatory factors. In favor of the latter hypothesis, Dorman *et al. *showed that neutrophil infiltration and liver injury occurred independent of CXC chemokine production in response to apoptotic cell injury in mice subjected to endotoximia [26]. The wild type as well as CXCR2 -/- mice showed similar neutrophil infiltration and liver injury. This hypothesis is supported by the data from the present study, which showed a significant increase in MIP-2 production and liver injury (as indicated by ALT and histopathology) by 6 h of reperfusion with moderate neutrophil infiltration into the ischemic liver lobe. At the peak of neutrophil infiltration (i.e. 24 h of reperfusion), MIP-2 levels were significantly attenuated, suggesting that neutrophils had infiltrated the liver parenchyma in response to the necrotic hepatocytes. Additionally, the notion that the neutrophil response is mediated by necrotic hepatocytes is further supported by the lack of neutrophil infiltration into the non-ischemic lobe of the IR mice where there is no hepatocellular damage. Of course, one should keep in mind a potential role of other factors such as reduced neutrophil deformability [27], sinusoidal cell swelling [28], and vasoconstriction of the sinusoids [29], which can facilitate sinusoidal neutrophil trapping. Other studies have underscored the role of complement factors as a mediator of IR injury [30,31]. A component of the complement system, C5a, can prime and activate Kupffer cells as well as neutrophils to generate ROS. Further, the complement system through formation of the membrane attack complex can potentially mediate IR injury. Evidence has been presented that the complement system is crucially involved in the pathogenesis of renal IR injury by modulation of neutrophil-independent, as well as neutrophil-dependent pathways [32]. The contribution of infiltrated neutrophils to hepatic IR injury and modulation of their functions by the cholinergic agonists in our study remains unclear at present and further studies are necessary to understand their potential role.

Hepatic IR injury is a complex process, and other inflammatory factors in addition to the cytokines are implicated in the pathogenesis. For example, studies have suggested a central role for ROS in the pathophysiology of liver IR injury [33,34]. The ROS generated during IR can induce apoptosis [35,36]. Additionally, the generated ROS can serve as a second messenger molecule in mediating TNF-α signaling and IL-8 secretion [37-39]. Further, studies have shown that hepatotoxic injury mediated by oxidative stress can be prevented by administration of anti-oxidants [40]. Whether cholinergic agonist treatment has any effect on ROS generation is of interest. It has been shown that in *in vitro *experiments, nicotine treatment inhibited the production of oxygen radicals by human neutrophils and macrophages and IL-1 by macrophages [41]. In contrast, a study by Jay *et al., *has shown that nicotine potentiates superoxide production by human neutrophils [42]. Iho *et al. *showed that nicotine induces human neutrophils to produce IL-8 (CXC chemokine) through the production of ROS and subsequent activation of NF-kB, with no effect on monocytes [43]. The discrepancy between these studies may be explained with respect to the cell type or their activation/differentiation status as well as the nicotine concentrations applied. In support of this notion, a study by Gillespie *et al., *has shown that high concentrations of nicotine attenuated formyl peptide-induced chemotaxis, while lower doses potentiated the response [44]. It is of interest to examine the effect of the cholinergic agonists on ROS production and the biochemical signaling in hepatic IR. Whether the cholinergic agonists protected the early phase of hepatic IR injury by attenuating a Kupffer cell-induced oxidant stress remains unclear, and further studies are necessary to examine its potential role.

The loss of protective effect of the cholinergic agonists during the late phase of liver IR remains unclear. One possible explanation may involve cholinergic receptor desensitization and/or recycling. Another possibility may be a lack of sufficient concentration of the agonists, due to their clearance from the body, and therefore, resulting in a diminished protective effect. This hypothesis was addressed by the further addition of doses of either agonist. There was no significant difference in liver IR injury after 6 and 24 h of reperfusion between the mice that received a single injection of the agonists prior to the onset of ischemia and those that received additional doses (2–4 injections) during the reperfusion. Further, the delay in hepatic injury may be due to the effect of cholinergic agonists on the hepatic microcirculation. The cholinergic receptors are present in hepatic microcirculation and have shown a role in vasodilatory function [45,46]. Other studies have shown the presence of non-neuronal nicotinic AChR on endothelial cells that were upregulated upon proliferation in response to hypoxia and ischemia [47]. Whether the cholinergic receptor agonists act on endothelial cell functions in hepatic IR remains to be elucidated.

In summary, the study presented indicates that AChR agonists provide a means to regulate cytokine production and to delay the hepatocellular injury caused by reperfusion of the ischemic liver. Modulation of the cholinergic anti-inflammatory pathway may have important therapeutic implications during the early phase of hepatic IR injury. Further studies are necessary to determine the exact role of the cholinergic system in liver IR injury during the early and late phases of reperfusion.

## Abbreviations used

**ACh**, acetylcholine; **AChR**, acetylcholine receptor; **DMPP, **1,1-dimethyl-4-phenyl L-pioperazinium iodide; **ELISA**, enzyme linked immunosorbent assay; **IR**, ischemia/reperfusion; **MIP-2**, macrophage inflammatory protein-2; **ROS**, reactive oxygen species; **TNF-α **, tumor necrosis factor-α.

## Competing interests

The author(s) declare that they have no competing interests.

## Authors' contributions

KP carried out the molecular analysis for PCR and real-time PCR, ALT measurements, and assisted in surgical procedures and collection of the tissue samples. JH carried out the histopathology evaluation. BU, JG, and RR participated in the design and development of the study, as well as preparation of the manuscript. EC conceived the study, participated in its design and coordination, carried out the surgical procedures and ELISA, data analysis and preparation of the manuscript. All authors read and approved the final manuscript.

## Pre-publication history

The pre-publication history for this paper can be accessed here:
